# Relevance of the Glycemic Index and Glycemic Load for Body Weight, Diabetes, and Cardiovascular Disease

**DOI:** 10.3390/nu10101361

**Published:** 2018-09-22

**Authors:** Sonia Vega-López, Bernard J. Venn, Joanne L. Slavin

**Affiliations:** 1 College of Health Solutions and Southwest Interdisciplinary Research Center, Arizona State University, Phoenix, AZ 85004, USA; 2 Department of Human Nutrition, University of Otago, Dunedin 9054, New Zealand; Bernard.Venn@otago.ac.nz; 3 Department of Food Science and Nutrition, University of Minnesota, St. Paul, MN 55108, USA; jslavin@umn.edu

**Keywords:** body weight, carbohydrates, glycemic index, glycemic load, glycemic response, satiety, type 2 diabetes, chronic disease risk

## Abstract

Despite initial enthusiasm, the relationship between glycemic index (GI) and glycemic response (GR) and disease prevention remains unclear. This review examines evidence from randomized, controlled trials and observational studies in humans for short-term (e.g., satiety) and long-term (e.g., weight, cardiovascular disease, and type 2 diabetes) health effects associated with different types of GI diets. A systematic PubMed search was conducted of studies published between 2006 and 2018 with key words glycemic index, glycemic load, diabetes, cardiovascular disease, body weight, satiety, and obesity. Criteria for inclusion for observational studies and randomized intervention studies were set. The search yielded 445 articles, of which 73 met inclusion criteria. Results suggest an equivocal relationship between GI/GR and disease outcome. The strongest intervention studies typically find little relationship among GI/GR and physiological measures of disease risk. Even for observational studies, the relationship between GI/GR and disease outcomes is limited. Thus, it is unlikely that the GI of a food or diet is linked to disease risk or health outcomes. Other measures of dietary quality, such as fiber or whole grains may be more likely to predict health outcomes. Interest in food patterns as predictors of health benefits may be more fruitful for research to inform dietary guidance.

## 1. Introduction

The appearance of glucose in the bloodstream following eating—the glycemic response (GR)—is a normal physiological occurrence that depends on the rate of glucose entry into the circulation, the amount of glucose absorbed, the rate of disappearance from the circulation due to tissue uptake, and hepatic regulation of glucose release [1]. Foods containing carbohydrates have a wide range of effects on the GR, with some resulting in a rapid rise followed by rapid fall in blood glucose concentrations, while others show an extended rise and slow extended fall in blood glucose. The Glycemic Index (GI) was created in 1981 as a tool for people with diabetes to select foods [2]. GI provides information on the GR that might be expected when a person consumes the quantity of a food containing a fixed amount of carbohydrate (usually 50 g). In this system, GR is defined as the increase in the blood glucose concentration following eating, expressed as the incremental area-under-the-blood-glucose-curve (iAUC) over a period of two hours. The GI value is actually given as a relative GR; the GR of the food is expressed as a percentage of the GR of a reference food (usually a glucose solution or white bread):

GI = (iAUC_test food_/iAUC_reference food_) × 100 (1)

To a large extent, control of the GR is governed by the amount of food eaten; that is, if a large amount of a low or a high GI food is consumed, the GR will be large and vice versa, a small amount of either a low or a high GI food will limit the GR. The concept of the glycemic load (GL) was introduced as a means of predicting the GR; it takes into account the GI and the amount of available carbohydrate in a portion of the food eaten (GL = GI × available carbohydrate in a given amount of food) [3].

Much work has been undertaken since the introduction of the concepts of GI and GL to ascertain how they relate to health and disease. In applying the concepts, foods have been classified by GI into low (GI ≤ 55), medium (GI 56–69), and high (GI ≥ 70) categories, and classified by GL as being low (GL ≤ 10), medium (GL 11–19), and high (GL ≥ 20). The GI and GL classification systems were developed arbitrarily in the sense that they did not relate to nutrient density of the food or to any risk factor for chronic disease as a consequence of consuming the food. The observational epidemiological work relating GI and GL to overweight and obesity, and to chronic disease risk has been controversial, as has been whether consuming diets with low GI translates into better health outcomes and more effective weight management for the general population. Therefore, the purpose of this review is to summarize the most recent evidence for short-term (e.g., satiety) and long-term (e.g., weight, cardiovascular disease, and type 2 diabetes) health effects associated with different types of GI diets.

## 2. Methods

Articles were initially selected by conducting a PubMed online search using the following keywords and combinations: glycemic index, glycemic load, diabetes, cardiovascular disease, body weight, satiety, and obesity. For purposes of this review, studies included were limited to those published in English between 2006 and 2018 conducted in adults and in which the study design allowed for a clear comparison between foods, meals, or diets with distinct GI (i.e., frank comparison of low GI foods, meals, or diets with their high GI counterparts). PubMed was last searched on 20 July 2018; data were extracted and summarized into tables for content review.

Cross-sectional studies were included if they had body weight or BMI, type-2 diabetes diagnosis, or a cardiovascular event as an endpoint. For intervention studies, only those with a randomized design were included in an attempt to assess evidence only from studies with greater internal validity than those using a quasi-experimental design. Studies aimed at assessing the effects of specific foods as part of a low GI diet (e.g., legumes or low GI fruits), those in which additional dietary components were part of one of the diet treatments (e.g., vinegar or olive oil), and those in which physical activity was part of the intervention were excluded because those additional components could have confounded the effects from the GI itself on study outcomes. Studies with a quasi-experimental design were not included. Animal studies were also excluded.

Additional considerations of inclusion criteria for studies incorporated in this review will be described within each section. Among the studies included herein, a majority focused on GI rather than GL because there has been no consensus of whether GI or GL is used in research settings. Descriptions of the studies were kept consistent with the parameter assessed (GI or GL).

**Figure 1:**
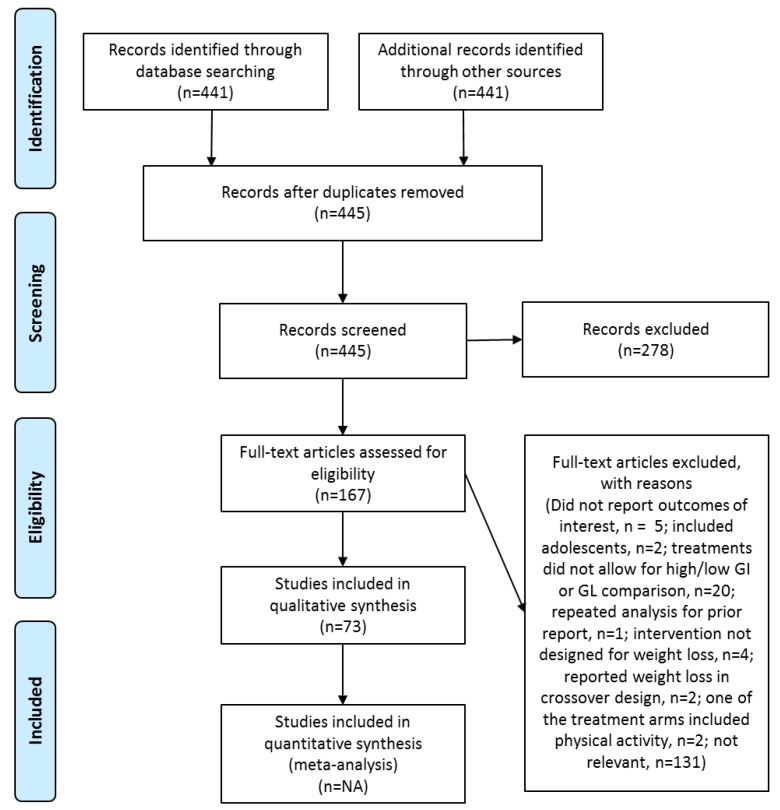
PRISMA Flow Diagram illustrating study selection.

Of the 445 articles retrieved from the keyword search, 278 were not relevant for this review. After reviewing the remaining 167 articles, 73 met the selection criteria and were included (Figure 1).

## 3. Glycemic Index/Glycemic Load and Satiety

Classification of foods based on their GI stems from the premise that the presence of different carbohydrates within foods elicits different GRs and potential downstream metabolic responses [2,4]. Regulation of satiety is a complex mechanism dependent on multiple factors, among them satiety-related hormones such as insulin, leptin, ghrelin, cholecystokinin (CCK), glucagon-like peptide 1 (GLP-1), peptide tyrosine-tyrosine (PYY), enterostatin, amylin, and oxyntomodulin [5,6]. Thus, there is no biomarker that could serve as a single measure of satiety. Although there is increasing interest in how different dietary components affect appetite or satiety, the specific effects of GI have not been thoroughly studied, and with a few exceptions, have been assessed using subjective self-reported measures. The most commonly used instruments for subjectively assessing satiety consist of visual analog scales that prompt a responder to rate their degree of satiety on a numerical scale graph (similar to a ruler) [7].

### 3.1. Acute Effects of Meals with Different GI

Studies that included an assessment of the effects of different GI meals on satiety have been designed to evaluate postprandial biomarker responses after acute ingestion of meals with different composite GI values. Although the standard methodology for assessing GI of a food involves measuring the GR over a 2-h period [8], there are no standard protocols for assessing the postprandial effects of different GI foods on other outcomes. Several studies have consisted of randomized crossover assessments of responses to breakfast meals after a standardized overnight fast, followed by a postprandial observation period of 2 to 4 h in length (Table 1) [9,10,11,12,13,14,15,16]. These interventions were conducted in healthy adults [10,13,15,16], adults with type 2 diabetes [11,12], pregnant women with gestational diabetes [9], and men with type 1 diabetes [14].

Although, as expected, low GI meals resulted in lower glycemic [9,10,11,12] and insulinemic [10] responses, subjective assessments of appetite, satiety, hunger, fullness, or desire to eat did not differ based on the GI of the test meal. Moreover, the lower glycemic and/or insulinemic responses observed in two of the studies were observed in the context of higher dietary fiber content of the meals [11,12]. One of those studies compared low and high GI meals with low and high fiber content and documented lower postprandial ghrelin responses only under the low GI/low fiber combination [11]. Similar to what was documented in the previously described studies, subjectively-measured satiety, appetite, or fullness were not different 12 h after consumption of macronutrient (protein, carbohydrates, and fat)—matched meals with low or intermediate GI in 12 adult Muslim men who were fasting during Ramadan [13]. Moreover, in a crossover study comparing responses to postexercise meals with low or high GI in individuals with type 1 diabetes, despite the greater postprandial glucose area under the curve with the high GI meal, there were no differences in glucose, insulin, glucagon, and glucagon-like peptide-1 (GLP-1) concentrations or subjective appetite ratings between meals [14].

Another approach to evaluating the short-term effects of the GI consists of monitoring responses to consecutive standardized meals with low or high GI over 10 to 12 h during different test days (Table 1). Two studies [15,16] that followed this approach included adult participants free of chronic conditions and reported responses as mean area under the curve for the entire follow-up period. In both studies, the amount of fiber consumed throughout the testing period was comparable. In a crossover study, 12 adult participants (23 ± 3 years old; 23.1 ± 1.9 kg/m^2^) were randomized to receiving meals with low or high GI and were monitored over 10 h, during which participants consumed four consecutive meals [15]. Insulin, cholecystokinin, and ghrelin responses were monitored over time. Although consuming the low GI meals induced lower glucose and insulin areas under the curve, there were no differences in cholecystokinin and ghrelin responses relative to consuming the higher glycemic index meals. In a separate study with 26 overweight or obese adults (44 ± 15 years old; 29.1 ± 1.8 kg/m^2^) participants were monitored over 12 h after consuming three consecutive low or high GI meals with low or high carbohydrate content [16]. The postprandial glucose and insulin areas under the curve were significantly greater only for the high GI/high carbohydrate combination, with no difference in subjectively assessed hunger among diets.

A study was undertaken to assess the satiety of 38 foods using the GI principle of expressing the satiating properties of a test food relative to a reference food of white bread [17]. In this scenario, a Satiety Index was created by providing servings of food containing a standardized energy intake of 1000 kJ (as opposed to a standardized amount of available carbohydrate for GI). The food with the highest SI was potato, a low energy dense food with a satiety rating of over three times that of white bread (SI 323%). Foods with lower SI tended to be high energy dense, low volume foods such as croissant (SI 47%) and cake (SI 65%). There was a positive relationship between serving weight and SI (*p* < 0.001). In effect, the food with the highest GI (potato) was the most satiating, and foods of smaller volume with lower GI (e.g., croissant and cake) were poorly satiating.

In summary, evidence regarding the short-term effect of the GI of foods or meals on satiety is drawn from randomized crossover studies that either assessed postprandial responses to a breakfast meal or monitored responses to consecutive meals over a 10 to 12 h period. Although the approach from these two types of studies is different, results have been consistent and do not support a short-term effect of the GI of foods or meals on satiety.

### 3.2. Effects of Chronic Intake of Diets with Different Glycemic Index

In order to assess the longer-term effects of GI on satiety, the approach has been to provide study participants with meals or diets differing in GI for an extended period of time varying from 4.5 days to 12 months, followed by an assessment of fasting biomarkers [18,19], postprandial responses to standardized meals [20,21], or physiological responses, including body weight [22,23,24] (Table 2).

In a crossover study, 40 adult women (20 White and 20 Black; ≥18 years old; 20 normal weight and 20 obese) consumed low or high GL diets for four days prior to a test day in which the 3-h postprandial responses to corresponding low or high GL meals were monitored [20]. The low GL diet resulted in lower glucose and insulin and higher ghrelin responses only among White participants, and no differences in self-reported desire to eat among all participants were documented. Fiber content of the diets was not reported. In contrast, a parallel study comparing the effects of consuming low or high GI diets for 10 weeks in 29 overweight women (31 ± 7 years old; 27.6 ± 1.5 kg/m^2^) documented an increase in self-reported fullness and a reduction in the desire to eat “fatty foods” among women allocated to the low GI diet group [21]. Biomarker responses were assessed after consumption of test breakfast meals with comparable amounts of fiber. Despite lower 4-h postprandial glucose and insulin responses among the low relative to the high GI group, there were no differences between groups in glucagon, leptin, ghrelin, or ad libitum energy intake after the test period.

Because altering the GI of all meals may be unattainable for some individuals, some have considered only modifying select components of the diet. A crossover intervention designed to assess the effects of consuming breakfast meals with different GI for 21 days included 21 overweight and obese adults (25–65 years old) [18]. Although the prescribed breakfast meals with low GI had greater fiber content, there were no differences in self-reported energy, macronutrient, or fiber intake between groups. Relative to the high GI breakfast period, participants had lower fasting glucose and reported greater satiety after consuming the low GI breakfast. No other differences in fasting biomarkers (insulin and lipids) were reported. In a separate randomized crossover intervention, 19 obese women (34–65 years old; 25–47 kg/m^2^) were assigned to ad libitum diets in which the GI was manipulated by prescribing use of the lower or higher GI versions of select carbohydrate-containing foods for 12 weeks [22]. Although with this approach all participants gained weight over time, there were no differences in changes in body weight, body composition, energy intake, or subjectively-assessed hunger or fullness based on the GI of the staples.

The long-term effects of consuming diets with low or high GI on satiety have been reported in three studies [19,23,24]. In a randomized parallel weight-loss feeding intervention (30% energy restriction), in which 34 overweight adults were given controlled diets with low or high GL, although participants significantly reduced body weight over time, there were no differences between groups in body weight or composition and self-reported hunger or satiety [23]. Similarly, in a 6-month weight-loss intervention in which 122 overweight or obese adults were randomized to two moderate carbohydrate diets with high- or low-GI or a low-fat/high GI diet, there were no differences in reported hunger or satiety among groups [24]. In a weight maintenance randomized crossover study with 40 normal weight and 40 overweight/obese men and women, there was no difference in hunger over the 4-week study period between diets but women (not men) reported feeling more full when consuming the low- compared with the high-GL diet [19]. Leptin concentrations were not significantly different after both diet periods.

In summary, studies reporting the effects of long-term consumption of diets with low or high GI have had inconsistent findings regarding the role of the GI on satiety. With a few exceptions, data were limited to subjective assessments of satiety, appetite, hunger, or fullness using visual analog scales. Among studies that reported ghrelin concentrations, results were generally null. Over the longer term, an association between GI or GL and satiety could manifest as having an effect on daily energy intake, and one might expect to observe a relationship between dietary GI or GL and energy intake in large-population-based observational work. However, energy intake was not different across categories of dietary GI or GL among a sample of 15,258 people across Europe [25], among 59,000 black women in the USA [26], among 74,248 US women, 90,411 US women, and 40,498 US men [27], or among 64,227 Chinese women [28].

## 4. Glycemic Index/Glycemic Load and Body Weight

### 4.1. Cross-Sectional Evidence Regarding the GI/GL and Body Weight

Evidence regarding an association between dietary GI or GL with body weight stems from several cross-sectional observations in diverse populations, including adults [29,30,31], young Japanese women (18–20 years old) [32], adults with type 2 diabetes [33,34,35], and older adults [36,37] (Table 3). Among different studies, the body-weight related outcomes most frequently reported were body mass index (BMI) and waist circumference. 

Findings regarding an association between GI or GL and body weight are equivocal. A cross-sectional analysis of 3931 Japanese young women (18–20 years old) reported positive associations between BMI and GI but not GL, suggesting that the type of carbohydrate-containing foods in the diet played a role in determining body weight [32]. Nevertheless, it is important to note that the difference in BMI between the highest and lowest quintile of BMI was relatively small (0.7 kg/m^2^). Among older adults, a study with 1152 participants (≥65 years old) reported no association between dietary GI and body weight or body mass index [36]. Similarly, a study with 343 participants from rural Spain (60–74 years old) reported no associations between dietary GI or GL and BMI or waist circumference, especially once diabetes status or hypoglycemic medications were included in the statistical model [37]. Further, a study 640 adults with type 2 diabetes reported no association between GL and body mass index [35]. In contrast, among 8,195 adults (35–74 years old) with a wide body mass index range (18.5–60 kg/m^2^), there was no association between GI and BMI and a negative association between GL and BMI [29]. Differences between the lowest and highest tertiles of GI or GL ranged between 0.7 and 1.0 kg/m^2^. For GI the negative association with body mass index was only statistically significant for women, not for men [29]. Whereas in larger-scale studies with healthy adults, there were no associations between GI/GL and waist circumference [30,31], the two smaller studies with type 2 diabetes patients (*n* = 175 and 238) reported a positive association between waist circumference and dietary GI [34] or GL [33].

Several studies also reported associations of body weight indicators with dietary fiber [29,32,34]. Among those, some reported a negative association between dietary fiber and BMI [32] or waist circumference [34].

In summary, cross-sectional data are inconsistent both in the direction and strength of association between GI or GL and body weight, and this being the case, do not support a strong role of dietary GI or GL on body weight. Among studies that reported an association between GI and body weight, the differences between extreme percentiles of BMI were small. Given that diets with lower GI often have greater fiber content, it is possible that any associations of GI with body weight are influenced by dietary fiber. These studies were not designed to assess whether specific sources of fiber (e.g., whole grains and fruits/vegetables) may impact body weight. An important limitation of cross-sectional studies is that GI or GL is calculated from self-reported diet data (often food frequency questionnaires).

### 4.2. Intervention Studies Assessing the Effects of GI/GL on Body Weight

For purposes of reviewing the effects of manipulating GI or GL on body weight, only studies designed as weight loss interventions were included (Table 4). These comprised eight studies ranging from 8 weeks to 18 months in duration that included overweight and obese adults [23,24,38,39,40,41,42,43]. Intervention studies with a crossover design were excluded from this review because fluctuations in body weight during an experimental period can confound results observed in a subsequent experimental phase. Studies focusing on weight maintenance after initial weight loss were also excluded.

Two studies reported significant differences in weight loss with low GI diets relative to the high GI diets [40,44]. In an 8-week intervention, 32 obese adults (36 ± 7 years old; 32.5 ± 4.3 kg/m^2^) were randomly assigned to follow one of two energy-restricted diets (−30% of energy expenditure) for 8 weeks with low GI (40–45) or high GI (60–65) [40]. Both groups lost a significant amount of weight relative to baseline (*p* < 0.001). However, participants in the low GI group lost significantly more weight than those in the high GI group (−7.5 vs. −5.3 kg, respectively; *p* = 0.032) and had significantly greater reductions in BMI (−7.6 vs. −5.4 kg/m^2^; *p* = 0.03). The lower GI diet was higher in fiber than the higher GI diet and the mean energy of the diets were 1495 ± 245 kcal/day and 1568 ± 225 kcal/day for the lower and higher GI diets, respectively.

In a separate study, 122 overweight and obese adults (30–60 years old; 27–35 kg/m^2^) were randomized to one of three energy-restricted diets (−500 kcal/day) for 6 months with moderate carbohydrate (~42% of total energy intake vs. the standard 55–60% of energy) and high GI, moderate carbohydrate and low GI, or low fat and high GI [24]. Although participants in the three groups experienced weight loss throughout the intervention, changes in BMI were greater after 12 weeks for the low GI than for the low fat group. There were no differences in changes in waist circumference of body composition among groups. Among participants who completed the 6-month study (*n* = 104), participants in the low GI diet had greater reductions in body mass index than those in the other two groups.

Four interventions compared energy restricted diets with low or high GI in adults at risk for heart disease (12-week intervention; *n* = 18; 35–65 years old; 27–35 kg/m^2^) [39], overweight adults (12-month intervention; *n* = 34; age 24–42 years old; 25–30 kg/m^2^) [23], adult women (18-month intervention; *n* = 203; 25–45 years old; 23–30 kg/m^2^) [41], and obese adults (3-month intervention; *n* = 40; 20–60 years old; 25–50 kg/m^2^) [38]. Two of these interventions were controlled-feeding studies in which all meals were provided to study participants [23,38]. Consistently, these studies reported weight reductions over time for all study participants, with no differences in body weight, BMI, or waist circumference changes between low or high GI groups.

Two additional controlled feeding weight loss studies compared diets differing in GI under either different degree of caloric restriction [42] or different carbohydrate content [43]. A 12-month intervention with 46 overweight adults (20–42 years old; 25–30 kg/m^2^) compared low and high GL diets with 10% or 30% energy restriction and reported no differences in body weight changes among groups [42]. Similarly, a 12-week weight loss intervention with 79 obese adults (45–65 years old; 28–38 kg/m^2^) compared low and high GI diets with moderate or high carbohydrate content and reported no differences in weight loss by GI or carbohydrate content of the diets [43]. This study further assessed metabolic adaptation 12 months after the weight loss period, and suggested no differences in weight regain based on GI during the weight loss phase.

From intervention studies, there is insufficient data to support a benefit from incorporating low GI alternatives to energy restriction for weight loss. Although some shorter-term studies suggested a benefit from lowering the GI of the diet for greater weight loss [40,44], highly-controlled feeding interventions suggested that manipulating the GI does not make a difference in weight-related outcomes [23,38,42,43].

## 5. Glycemic Index/Glycemic Load and Cardiometabolic Disease Risk

Evidence regarding associations between GI or GL and cardiometabolic disease risk will be separately described first for type 2 diabetes, followed by cardiovascular disease risk, and then risk factors. Cardiovascular disease is a complex condition with diverse contributing factors including those associated with inflammation and oxidative stress. For purposes of this review, only studies that reported traditional cardiovascular disease risk factors (lipids, blood pressure, or C-reactive protein) were included. Reports with more specific inflammatory and oxidative stress markers are scarce thus limiting the ability to draw conclusions regarding existing evidence of the effect of the GI on those outcomes.

### 5.1. Epidemiological Evidence Regarding GI/GL and Markers of Glucose Homeostasis

#### 5.1.1. Cross-Sectional Studies

The cross-sectional analyses that contribute to the evidence regarding an association between GI or GL and markers of glucose homeostasis (fasting plasma glucose, 2-h blood glucose, glycosylated hemoglobin (HbA1c), or calculated indices of insulin sensitivity such as the homeostatic model assessment of insulin resistance (HOMA-IR)), included adults with [33,35] or without [30,31,45,46,47,48] known type 2 diabetes. Notably, only one report included individuals with a range of glycemic control status (healthy, insulin resistance, and type 2 diabetes) [49] (Table 5).

In studies that included individuals with insulin resistance and diabetes, results were inconsistent. Using baseline data from an intervention among 238 obese low-income Latino adults with type 2 diabetes, GI, but not GL, was positively associated with HbA1c [33]. In an analysis of data from 640 adults with type 2 diabetes, fasting glucose and HbA1c were positively associated with GL, but not GI after adjusting for multiple potential dietary confounders [35]. In this study, HbA1c was also positively associated with total carbohydrate intake. In contrast, an analysis from the Insulin Resistance Atherosclerosis Study with 1255 adults with or without insulin resistance or diabetes reported no associations between GI or GL with fasting glucose, 2-h glucose, or HbA1c [49].

Although several studies among individuals without type 2 diabetes pointed to an association between GI or GL with markers of glucose homeostasis, some findings were still inconsistent. Among 2078 Inuit adults, logistic regression analyses suggested positive associations between GI and fasting glucose and between GL and insulin resistance (HOMA-IR) after adjustment for confounders [45]. No associations were documented for GI with 2-h glucose, HbA1c. or HOMA-IR, or for GL with fasting glucose, 2-h glucose, or HbA1c. In a study with 668 adults from the Canary Islands, insulin resistance (HOMA-IR) was positively associated with GL, but this association lost its statistical significance when fructose intake was added to the model [47]. This suggests that fructose intake plays a role in insulin resistance that is not captured by measuring the GI or GL of the diet.

Results from a study with 2457 adults indicated positive associations of GL with fasting glucose and 2-h glucose but only among non-obese individuals (BMI < 30 kg/m^2^) [30]. In this study no associations between GI and fasting glucose or 2-h glucose were documented. In the Framingham Offspring Study, dietary GI was positively associated with fasting insulin after, but not with fasting glucose [31]. A study with 878 postmenopausal women reported no associations between GL and fasting glucose, insulin, or HOMA-IR [48]. Finally, a cross-sectional analysis with 3931 Japanese young women (18–20 years old) indicated positive associations of dietary GI with fasting glucose and HbA1c and of GL with fasting glucose [46].

In summary, results from cross-sectional studies relating GI or GL with markers of glucose homeostasis or insulin resistance are inconsistent. Studies are variable in design, data used to calculate the exposure of interest (GI/GL), and adjustments used for the statistical analysis of the data. The loss of statistical significance when models were adjusted for potential dietary confounders, such as fiber or fructose, suggests that carbohydrate-related factors other than GI and GL may play a role in glycemic control. Furthermore, diet data from which GI or GL were calculated in cross-sectional studies is based on self-report, mostly from food frequency questionnaires, limiting the validity of the data. At present, data do not support a reliably robust association between dietary GI or GL and markers of glucose homeostasis or insulin resistance.

#### 5.1.2. Prospective Studies

Evidence published prior to 2006 regarding the association between dietary GI or GL with type 2 diabetes risk from large prospective studies provided inconclusive findings. Dietary GI was positively associated with type 2 diabetes risk in the Nurses’ Health Study, the Health Professionals Follow-Up Study, and the Melbourne Collaborative Study [3,50,51,52]. GL was also positively associated with type 2 diabetes risk in the Nurses’ Health Study [3]. However, no associations between GI or GL were documented from the Atherosclerosis Risk in Communities Study [53]. More recent publications of prospective studies assessing type 2 diabetes risk based on dietary GI or GL included studies with adult women [28,54], adult men [55,56], women and men combined [25,57,58,59,60], and older adults [61] (Table 6).

Several studies reported increased risk of type 2 diabetes diagnosis with higher dietary GI or GL. In a 20-year follow-up of the Nurses’ Health Study including 85,059 women, the relative risk (RR) of type 2 diabetes diagnosis was greater with increased dietary GL (RR = 2.47; 95% CI 1.75–3.47) after adjusting for potential confounders including dietary fiber [54]. Low carbohydrate intake was associated with increased risk of type 2 diabetes (RR = 1.26; 95% CI 1.07–1.49). Type 2 diabetes risk by dietary GI was not reported. In a 4.6-year follow-up of 64,227 middle-aged Chinese women in the Shanghai Women’s Health Study, type 2 diabetes risk was significantly higher among participants in the highest quintile of carbohydrate intake (RR = 1.28; 95% CI 1.09–1.50), GI (RR = 1.21; 95% CI 1.03–1.43), GL (RR = 1.34; 95% CI 1.13–1.58), and common staple consumption (mainly rice, noodles, steamed bread, and other bread; RR = 1.37; 95% CI 1.11–1.69) [28]. When participants were stratified by waist-to-hip ratio or BMI, GI was associated with higher diabetes risk only among overweight or obese participants. In this study, dietary fiber or its potential sources (e.g., whole grains and fruit/vegetables) were not considered in the analysis. In a 6-year follow-up of 1995 middle-aged Japanese men, type 2 diabetes risk was greater with increased GI (Hazard Ratio (HR) = 1.96; 95% CI 1.04–3.67), but not with increased GL, energy intake, or fiber intake [55]. Type 2 diabetes risk was also greater with increased GL (HR = 1.27; 95% CI 1.11–1.44) among 37,843 adults participating in the European Prospective Investigation into Cancer and Nutrition (EPIC) Study in the Netherlands [59]. In this study the association for GI and diabetes risk was only borderline significant (HR = 1.08; 95% CI 1.00–1.17). Moreover, the risk of diabetes was significantly greater with increased carbohydrate intake (HR = 1.20; 95% CI 1.01–1.42) and lower with increased fiber intake (HR = 0.89; 95% CI 0.82–0.98).

In contrast to the previous reports, six studies did not find an association between GI or GL and type 2 diabetes risk. These included a 4-year follow-up of 1898 older adults participating in the Health ABC study [61], a 10-year follow-up with 2123 Australian adults [57], a 13-year follow-up of 7321 Caucasian adults from the Whitehall II study [58], a 12-year follow-up of 4093 Dutch adults [60], a 12-year follow-up of 25,943 male smokers [56], and a 12-year follow-up in a random subcohort of the European Prospective Investigation into Cancer and Nutrition Study [25].

In summary, results from prospective studies continue to suggest an equivocal association between type 2 diabetes risk and GI or GL. Type 2 diabetes risk appears to have a stronger association with GL than with GI, but dietary fiber or its sources (e.g., whole grains and fruits/vegetables), and total carbohydrate intake could contribute to the array of results reported. Although these observations are generally derived from large-scale prospective studies, a limitation is that dietary GI and GL often derived from self-reported data generally obtained using food frequency questionnaires.

### 5.2. Intervention Studies Assessing the Effects of GI/GL on Markers of Glucose Homeostasis

Several dietary interventions were designed to compare low GI or GL diets with their high GI or GL counterparts using crossover [18,62,63,64,65] or parallel [24,38,66,67,68,69,70] randomized designs (Table 7). Among these studies, those with a crossover design were shorter in duration (10 days to 5 weeks per intervention phase), whereas parallel design interventions ranged from 45 days to 12 months in duration. Studies included adults with type 2 diabetes [66,67,68,70], healthy adults with diverse weight status [65], or overweight and/or obese but otherwise healthy adults [18,24,38,62,63,64,69]. Reports from interventions involving nutrition education in which the use of GI was compared to current dietary recommendations for patients with type 2 diabetes (e.g., American Diabetic Association guidelines [71]) were excluded from this review because participants in different treatment arms were given different diet recommendations, which resulted in diets with multiple incomparable factors aside from the GI or GL of the diet.

Four of the studies with a crossover design were well-controlled feeding interventions in which overweight or obese participants received all meals during the test periods in adequate amounts for weight maintenance [62,63,64,65]. In the shortest of these controlled studies, 12 overweight or obese young adults (18–35 years old) received low- or high-GI diets for 10 days each in random order [62]. Although insulin sensitivity, assessed using a frequently sampled intravenous glucose tolerance test at the end of each phase, was greater at the end of the low GI diet, there were no differences in response between interventions. In a longer intervention, 24 overweight or obese men (34.5 ± 8.1 years old; 27.8 ± 3.5 kg/m^2^) were provided with low or high GI diets for four weeks in random order [64]. Although participants experienced reductions in both fasting glucose and insulin with both diets relative to baseline, changes were not significantly different between diets. In contrast, an intervention with 80 healthy adults (29.6 ± 8.2 years old; 27.4 ± 5.9 kg/m^2^) resulted in lower fasting glucose and insulin-like growth factor 1 (IGF-1) concentrations after consuming a low GL diet for four weeks, relative to a high GL diet [65]. When analyzing separately based on participant body fat, the effect of the low GL diet on fasting glucose concentrations were only significant among individuals with high body fat (≥25% for males or ≥32% for females). Fasting insulin concentrations and HOMA-IR index were not different between diet phases. In a multisite study 163 overweight adults (53 ± 11 years old; 32 ± 6 kg/m^2^) were provided with four different controlled diets for five weeks in random order: high carbohydrate/high GI, high carbohydrate low GI, low carbohydrate high GI, and low carbohydrate low GI [63]. Consumption of a low GI diet resulted in a lower insulin sensitivity index than the high GI diet only in the context of high carbohydrate intake (~58% of energy), with no differences observed when the diets had a low carbohydrate content (~40% of energy). In the only crossover study in which participants were not provided with all meals, 21 overweight or obese adults (25–65 years old) were given breakfast replacements with different GI for 21 days [18]. Participants had lower fasting glucose after the low GI breakfast replacement phase (88 ± 2 mg/dL) than after the high GI breakfast replacement phase (92 ± 3 mg/dL; *p* < 0.05). However, there were no differences in fasting insulin concentrations or HOMA index.

Two parallel controlled feeding interventions compared energy restricted diets with low or high GI in obese adults (3-month intervention; *n* = 40; 20–60 years old; 25–50 kg/m^2^) [38] or overweight and obese adults (6-month intervention; *n* = 122; 30–60 years old; 27–35 kg/m^2^) [24]. Although these studies reported significant reductions in fasting glucose [38] or HOMA index [24,38] over time, changes in these markers of glucose homeostasis were not different between diets. Moreover, HbA1c did not change with either diet. Two smaller-scale parallel studies of shorter duration involved randomizing overweight or obese adults to either a low GI diet or its high GI counterpart for 30 days (*n* = 20, all with type 2 diabetes; 18–55 years old; 29.2 ± 4.8 kg/m^2^) [70] or 45 days (*n* = 19; 22–38 years old; 27–35 kg/m^2^) [69]. Participants consumed two of their daily meals in the laboratory, with the rest of their food consumed under free-living conditions following recommendations for food selection based on GI lists. In the shorter of these studies, participants in the high GI diet had a significant increase in fructosamine concentrations relative to baseline, but other biomarkers assessed (glucose, lipids, adiponectin, and CRP) did not change [70]. In the longer of these two studies, although participants in the low GI diet experienced small reductions in waist circumference and body fat relative to baseline, there were no differences in glucose, insulin, or leptin responses between groups [69].

The two longer-term studies conducted in patients with type 2 diabetes had a parallel design and yielded conflicting results [66,67,68]. In a 6-month intervention, 210 participants (HbA1c 6.5–8.0%; using hypoglycemic medications) were randomized to follow either a low GI diet or a high-cereal fiber diet that included cereal-based sources of fiber (e.g., whole wheat foods and brown rice) [66]. As part of the intervention, participants received dietary advice to comply with the diets. At the end of the intervention period, participants in the low GI group consumed a diet with 18.7 g of fiber per 1000 kcal and a GI of 69.6, whereas those in the high fiber group consumed a diet with 15.7 g of fiber per 1000 kcal and a GI of 83.5. Relative to baseline values, all participants had lower fasting glucose and HbA1c concentrations at the end of the study, but those following the low GI diet had a greater decrease in HbA1c (−0.50% with the low-GI diet vs. −0.18% with the high-cereal fiber diet; *p* < 0.001) and fasting plasma glucose (−8% with the low-GI diet vs −3% with the high-cereal fiber diet; *p* = 0.02).

In a separate 12-month intervention, 162 participants (HbA1c ≤ 130% upper normal limit; BMI 20–40 kg/m^2^; without medications) were randomized to follow a high-carbohydrate/high-GI diet, a high-carbohydrate/low-GI diet, or a low-carbohydrate/high-monounsaturated fat diet [67,68]. Participants received counseling by a dietitian to follow the prescribed diets and were provided different key foods to consume with the low or high GI diets. At the end of the intervention participants in the three groups reported comparable energy intake, but dietary fiber was significantly higher among participants in the low GI diet (37 ± 1.5 g/day) than among those following the high GI diet (21 ± 0.8 g/day) or the low carbohydrate diet (23 ± 0.8 g/day), mainly because the key foods provided in the low GI diet were naturally higher in fiber than the key foods provided in the other two diets. Although HbA1c and fasting glucose improved within the first three months of the intervention with the low GI diet, there were no differences among groups in these markers of glycemic homeostasis at the end of the intervention, and in fact HbA1c significantly increased in all groups relative to baseline concentrations (~0.2% from baseline; *p* < 0.0001) [68]. Moreover, participants in the low GI or low carbohydrate diet groups had a small, but significant, increase in fasting plasma glucose over time (~5% from baseline; *p* < 0.0001) [67]. Participants in the low GI diet group had the lowest 2-h glucose and a higher disposition index, an indicator of β-cell function, relative to the low-carbohydrate diet (*p* = 0.036).

In a systematic review and meta-analysis of the effects of dietary GI on glycemic control in people with type 2 diabetes, the authors concluded that a low GI diet is effective at lowering fasting blood glucose and HbA1c relative to a comparison diet [72]. This conclusion appears to be at odds with the findings reported here, but note that the meta-analysis was restricted to five studies carried out in people with type 2 diabetes and the findings were largely influenced by two of those studies, one of which was excluded from our review [73]. In that study, the low GI diet was achieved through the use of legumes, and the comparison diet contained more fiber (presumably soluble), less carbohydrate, less saturated fat, and more plant protein—factors that could contribute to the outcome independent of differences in GI.

In summary, although some intervention studies suggested favorable effects of low GI diets on fasting glucose, findings regarding effects on insulin, insulin sensitivity, or HbA1c are equivocal. In some studies, the benefit of a low GI diet may have been associated with higher fiber or lower carbohydrate consumption. Improvements in markers of glucose homeostasis were also observed in the context of energy restriction (in weight loss studies).

### 5.3. Epidemiological Evidence Regarding GI/GL and Cardiovascular Disease Risk Factors

#### 5.3.1. Cross-Sectional Studies

The cross-sectional association between GI or GL and cardiovascular disease risk factors has been assessed in several cross-sectional studies (Table 8) [30,31,36,46,48,74,75,76]. These studies included a combination of adult men and women [30,31,75,76], adult women [46,74], postmenopausal women [48], and older adults [36,37].

Results regarding the association of GI or GL and blood lipids are mixed. Regarding total- and LDL-cholesterol, most studies have failed to find an association with GI [30,31,36,37,46,75] or GL [37,48]. One of the exceptions is a study in which women in the highest quintile of dietary GI had significantly higher total- and LDL-cholesterol concentrations by approximately 2 mg/dL relative to those in the lowest quintile [74]. Moreover, positive associations with total- and LDL-cholesterol were reported when the GL was used in the analysis instead of the GI Liese, Gilliard, Schulz, D’Agostino, and Wolever [75].

Although some studies have failed to find an association between dietary GI and HDL-cholesterol [36,37,46,75], some studies have documented that individuals in the highest tertile or quintile of GI have significantly lower HDL-cholesterol concentrations relative to those in the group with the lowest GI [30,31,74]. In these studies the reported HDL-cholesterol concentration difference between individuals in the extreme quintiles of GI has been small, ranging between 2 and 4 mg/dL. Some studies have also documented associations between triglyceride concentrations and dietary GI [30,31,46,74] or GL [48], with differences between extreme percentiles ranging from 11 to 21 mg/dL.

Evidence regarding the association of dietary GI with other cardiovascular disease risk factors is limited. Among studies that included results based on blood pressure, no significant associations with GI were reported [30,36,37]. Only one study conducted in adult women reported a significant positive association between dietary GI and C-reactive protein [74]. Differences between extreme quintiles were of small magnitude (0.21 mg/L based on adjusted values). A study that included men aged 55 to 80 years and women aged 60 to 80 years, with and without type 2 diabetes, suggested that greater dietary GI, but not GL, is associated with greater prevalence of metabolic syndrome in individuals <75 years old without diabetes, and with hypertriglyceridemia among individuals 65 to 74 years without diabetes [76]. GI or GL were not associated with other components of the metabolic syndrome. No associations were found among participants with type 2 diabetes.

In summary, findings from cross-sectional studies generally do not support an association between dietary GI or GL and blood lipid concentrations. Studies that reported such associations documented differences in LDL- or HDL-cholesterol concentrations of small magnitude and questionable physiological effect when comparing extreme percentiles of GI. Nevertheless, these small differences may have important implications at the public health level. The evidence regarding the association of dietary GI with other cardiovascular disease risk factors is limited.

#### 5.3.2. Prospective Studies

Several prospective studies assessing the relationship between dietary GI or GL and cardiovascular disease risk were identified (Table 9). Those reporting on cardiovascular disease mortality [77,78] or on incident cardiovascular diseases (all combined) [79,80], coronary heart disease [81,82,83,84,85], stroke [80,82,85], myocardial infarction [80,86], or heart failure [87] were included herein. Most studies provided risk estimates that were adjusted for dietary factors, age, body mass index, and other potentially confounding factors. Meta-analyses were not included because they generally combined outcomes in their analysis [88].

The majority of analyses have focused on incident coronary heart disease [81,82,83,84,85]. Of these studies, those that reported no significant association between GI and incident coronary heart disease included an 11.9-year follow-up of 8855 men and 10,753 women [82], a 7.9-year follow-up of 44,132 adults [84], and a 9.8-year follow-up of 117,366 Chinese adults [81]. In this last study, coronary heart disease risk was positively associated with GL (HR = 1.87; 95% CI 1.00–3.53), refined grains intake (HR = 1.80; 95% CI 1.01–3.17), and total carbohydrate intake (HR = 2.88; 95% CI 1.44–5.78). Notably, about 68% of energy was provided by dietary carbohydrate in this population. A separate 17-year follow up of 13,051 White and African American adults reported an increased risk for coronary heart disease only among African American individuals (HR = 1.16; 95% CI 1.01–1.33) [83]. However, this association was no longer significant when individuals with diabetes were excluded from the analysis. In the same study, the significant association between coronary heart disease risk and GL was only significant among Whites (HR = 1.11; 95% CI 1.01–1.21), but the association was no longer significant when individuals with diabetes were excluded. Finally, a 9-year follow-up of 15,714 Dutch women resulted in significant associations of incident coronary heart disease with GI (HR = 2.88; 95% CI 1.44–5.78), but not GL [85].

The relationship between dietary GI or GL with incident cardiovascular diseases (all combined) was assessed in a 6-year follow-up of Swedish men 45–79 years of age [80] and in a subsequent analysis of 4167 participants from the same study with established cardiovascular disease [79]. Both analyses resulted in no significant associations. Additional analyses resulted in no significant associations of GI or GL with risk of stroke [80,82,85], myocardial infarction [86], or heart failure [87].

Two studies assessed the relationship between GI or GL and cardiovascular disease mortality. In a 9.2 year follow-up among 6192 adults with type 2 diabetes, neither GI nor GL were associated with risk of cardiovascular mortality [78]. Furthermore, greater fiber intake was associated with lower risk for cardiovascular mortality (HR = 0.76; 95% CI 0.64–0.89). Similarly, a 16-year follow-up of 28,356 Japanese adults did not result in an association between GL and cardiovascular mortality [77]. The association between GI and cardiovascular mortality was only significant in women (HR = 1.56; 95% CI 1.15–2.13).

From prospective studies, the evidence regarding the association between dietary GI or GL and cardiovascular disease incidence or mortality is equivocal and may suggest that other dietary factors such as fiber and total carbohydrates may play a role. Although these observations are generally derived from large-scale studies, a limitation is that dietary GI and GL often derived from self-reported data generally obtained using food frequency questionnaires.

### 5.4. Intervention Studies Assessing the Effects of GI/GL on Cardiovascular Disease Risk Factors

The long-term effects of consuming diets with low or high GI have been assessed in several randomized interventions (Table 10). Most studies with a crossover design were well-controlled feeding studies in which overweight or obese participants received all meals during the test periods in adequate amounts for weight maintenance [62,63,64,89].

The shortest of these interventions provided the controlled meals to 12 overweight or obese young adults (18–35 years old) for 10 days and reported no differences in fasting lipids or C-reactive protein at the end of the low or high GL diet periods [62]. In contrast, consumption of a low GI/lGL diet for four weeks increased total- and LDL-cholesterol concentrations by approximately 8 and 6 mg/dL, respectively, among 24 overweight or obese adults [64]. In this study, consumption of the high GL diet reduced total-, LDL-, and HDL-cholesterol concentrations by approximately 14, 13, and 4 mg/dL, respectively. No changes in C-reactive protein and other inflammatory markers occurred with either of the diets. Similarly, in a multisite study with 163 overweight adults, consumption of low GI diets for five weeks resulted in increased LDL-cholesterol concentrations relative to the high GI diet period by approximately 6% (from 139 to 147 mg/dL), but only in the context of high carbohydrate intake (~58% of energy) [63]. No differences in lipid responses to low or high GI diets were observed when the diets had a low carbohydrate content (~40% of energy). There were no effects on other markers of cardiovascular disease risk, including HDL-cholesterol, triglycerides, and blood pressure. In a different feeding study with 80 overweight or obese adults (18–45 years old), consuming a low GL diet for four weeks resulted in lower C-reactive protein concentrations by approximately 0.24 mg/L relative to the high GL diet phase but only among obese individuals [89]. No effects were reported for other inflammatory cytokines, and lipids were not reported. The only crossover study in which participants were not provided with all meals was a 21-day intervention in which 21 overweight or obese adults were given breakfast meals with different GI [18]. No differences in fasting lipids were reported.

Some of the parallel design studies reporting the effects of GI of the diet on cardiovascular disease risk factors were designed as weight loss interventions ranging from 12 to 24 weeks in duration for overweight and obese adults [38,39,90,91]. In the only controlled feeding intervention in which all meals were provided to study participants, 40 obese adults with at least two components of the metabolic syndrome were assigned to low or high GI hypocaloric diets for 12 weeks [38]. Weight loss was comparable in both groups, and was accompanied by reductions in waist circumference, fasting glucose and HOMA index, total- and LDL-cholesterol, triglycerides, and systolic blood pressure, all of which were not different between groups. The only differential response observed was on endothelial function assessed by flow mediated dilation (FMD), a method that measures the changes in arterial wall dilation after a stimulus. Relative to baseline values, FMD increased 2.3% in the low GI diet group, but decreased 1.0% with the high GI diet, suggesting an improvement in endothelial response with the low GI diet. In the other two parallel design interventions, participants (35–65 years old; 27–35 kg/m^2^) were randomized to low or high GL diets and were given advice on energy restriction, weight loss, and how to follow the assigned diet [39,90]. No meals were provided to the participants.

In the 12-week study conducted with 13 healthy adults, the greater weight loss observed with the low GL diet was not accompanied by significant changes in total-, LDL- or HDL-cholesterol or triglycerides [39]. In contrast, a 6-month intervention with 38 men with increased cardiovascular risk resulted in lower total cholesterol and ambulatory 24-h blood pressure among participants in the low GL diet than those following the high GL diet. No differences between groups were observed for other lipids. Finally, a 6-month intervention in which 773 overweight or obese adults (18–65 years) were assigned to LGI or HGI diets (either low or high in protein) reported that consumption of the low GI diets resulted in a reduction in hsCRP concentrations [91]. No other cardiovascular disease risk factors were reported.

Two parallel design interventions that assessed cardiovascular disease risk factors, and in which test diets were prescribed without energy restriction to study participants, were identified [92,93]. In a 5-week intervention with 38 adults (20–60 years old; 25–30 kg/m^2^), participants were randomized to receiving low or high GI starchy foods to incorporate ad libitum with the rest of their diets [92]. At the end of the intervention, participants in the low GI foods group had a significantly greater decrease in weight and body fat mass accompanied by greater, albeit not significantly different, reductions in total and LDL-cholesterol concentrations. In a 12-week intervention, 129 overweight or obese young adults (18–40 years old; 25–30 kg/m^2^) were randomized to one of four low-fat diets: high GI/high carbohydrate, low GI/high carbohydrate, high GI/high protein, or low GI/high protein [93]. Participants received sample menus to be used as guide to follow the assigned diets. Relative to baseline, all groups lost weight, with no differences among groups. No differences in lipids were reported, with the exception of LDL-cholesterol, which was significantly greater by about 10 mg/dL at the end of the study for participants consuming the high GI/high protein diet.

In summary, intervention studies that assessed cardiovascular disease risk factors in response to diets with different GI have also provided conflicting results. In two well-controlled crossover feeding trials of at least four weeks in duration, consumption of low GI or GL diets was associated with less favorable LDL/HDL profiles than their high GI or GL counterparts in the context of high carbohydrate diets. In parallel design studies, most of the findings point to the lack of effect of dietary GI or GL on cardiovascular disease risk factors.

## 6. Conclusions

As outlined in this review of the literature, findings reported over the past decade regarding the clinical utility of the GI for these outcomes are equivocal, consistent with earlier reviews [94,95]. The variety in findings probably depend on a complex interplay between different factors associated with issues related to dietary factors influencing carbohydrate digestion and metabolism (e.g., dietary fiber or amount of carbohydrate in the diet), diversity in study design and study populations, and limitations associated with different study designs. Moreover, outcome measures reported in the studies included herein could have been influenced by other factors not assessed by these studies. For example, studies that controlled for fiber only took total amounts into consideration but not individual food sources such as whole grains, fruit/vegetables, etc. Moreover, the possibility that different GI foods/diets may impact gut microbiome composition, and therefore have distinct downstream metabolic effects related to the outcomes reported in this report, cannot be ruled out.

A particular issue noted is the fact that most data regarding the clinical implications of dietary GI have been derived from observational studies. Given the limitations of dietary assessment in those studies (mostly self-reported data from questionnaires that were not designed and have not been validated to test for GI and GL), there is a need for more highly controlled feeding interventions to test whether diets with different types of carbohydrates indeed elicit different metabolic effects. It is noteworthy that in intervention studies the observed effects of lower GI diets on body weight and markers of glucose homeostasis and CVD risk, when present, are generally of small magnitude. Although this may be beneficial at the public health level, the clinical impact at the individual level is questionable. Moreover, future research regarding the effects of different foods on satiety should focus more on the physiological responses rather than subjective measures.

The use of the GI for clinical guidance also warrants further consideration. At the public level, the concepts of GI and GL are generally misunderstood [96]. Moreover, the large intra- and interindividual variability in glycemic responses to a food [97], coupled with the diversity of GI values reported for some comparable foods [98], suggests that making dietary recommendations based on GI may be misleading, especially since low GI does not always mean high nutritional value, and high GI foods, such as potato, may have other healthful qualities including low energy density and a high satiety rating [99]. Thus, focusing on overall dietary quality and promoting the healthful aspects of the diet (e.g., dietary fiber and fruit and vegetable intake) may be a better approach to help reduce chronic disease risk.

## Figures and Tables

**Table 1:** Intervention studies assessing the acute effect of low GI foods or meals on appetite/satiety ^1^.

Study	Sample	Design	Duration	Intervention	Treatment Effects (Low vs. High GI)	Greater Fiber (Low GI)
Louie et al. [9]	10 women w/GDM 30–32 weeks gestation 18–45 years	R; X	2 h PP responses to breakfast meal	LGI HGI	↓ PP Glucose ↔ Satiety (subj)	No
Makris et al. [10]	16 sedentary adults 38 ± 11 years 30.9 ± 3.7 kg/m^2^	R; X	4 h PP responses to breakfast	HGI-Hprot HGI-Lprot LGI-Hprot LGI-Lprot	↓ Glucose, insulin No protein effects No GI × protein effects ↔ Ad libitum energy intake ↔ Hunger (subj) ↔ Satiety (subj)	No
Silva et al. [11]	14 adults w/T2D 66 ± 5 years 27.2 ± 3.1 kg/m^2^ HbA1c: 6.6 ± 0.9%	R; X	3 h PP responses to breakfast meal	HGI-HF HGI-LF LGI-HF LGI-LF	↓ Glucose LGIHF vs. HGILF ↓ Ins HGIHF vs. HGILF ↓ Ghrelin LGIHF @ 180 min ↔ Appetite (subj)	NA
Lobos et al. [12]	10 obese adults w/T2D and intensive insulin therapy 55 ± 6 years 34.7 ± 2.4 kg/m^2^	R; X	2 h PP responses to breakfast meal	LGI breakfast HGI breakfast	↔ Satiety (subj) ↓ Glucose	Yes
Png et al. [13]	12 Muslim adult men 28 ± 7 years 51 ± 9 kg	R; X	12 h PP after ingestion of the last meal before fast during Ramadan	LGI (37) Control (GI ~57)	↔ Satiety, appetite, fullness (all subj)	NR
Campbell et al. [14]	10 men with T1D 27 ± 1 years 25.5 ± 0.03 kg/m^2^	R; X	Postprandial responses to postexercise meal	LGI meal HGI meal	↓ Glucose AUC ↔ Glucose, insulin, glucagon, GLP-1 ↔ Appetite (subj)	No
Reynolds et al. [15]	12 adults 23 ± 3 years 23.1 ± 1.9 kg/m^2^	R; X	10 h PP responses to 4 consecutive meals	Low-GI diet High-GI diet	↓ Glucose, insulin ↔ CCK, ghrelin	No
Liu et al. [16]	26 overweight/ obese adults 44 ± 15 years 29.1 ± 2.8 kg/m^2^	R; X	PP responses over 12 h including std breakfast/lunch/dinner	HGI-HCHO HGI-LCHO LGI-HCHO LGI-LCHO	↓ Glucose all vs. HGI-HCHO ↓ Insulin all vs. HGI-HCHO ↔ Hunger (subj)	No

^1^ Abbreviations: GDM: gestational diabetes mellitus; GI: glycemic index; HCHO: high carbohydrate; HF: high fiber; HGI: high GI; Hprot: high protein; LCHO: low carbohydrate; LF: low fiber; LGI: low GI; Lprot: low protein; NA: not applicable; NR: not reported; PP: postprandial; R: randomized; subj: subjective measure; T1D: type 1 diabetes; T2D: type 2 diabetes; X: crossover design; ↓ lower; ↔ no difference.

**Table 2:** Intervention studies assessing the effects of chronic intake of diets with different GI on appetite/satiety ^1^.

Study	Sample	Design	Duration	Intervention	Treatment Effects (Low vs. High GI)	Greater Fiber (Low GI)
Pal et al. [18]	21 overweight/obese adults Age: 25–65 years	R; X	21 days	LGI breakfast replacement HGI breakfast replacement	↓ Glucose ↔ TG, insulin, LDL-C, HDL-C ↑ Satiety (subj)	Yes for breakfast replacement No for overall diet (self-reported)
Chang et al. [19]	80 overweight/obese adults 18–45 years 27.5 ± 5.9 kg/m^2^	R; X	4 weeks	LGL diet HGL diet	↑ Satiety (subj) ↓ Food cravings ↔ Leptin	Yes
Brownley et al. [20]	40 women 20 normal weight, 20 obese ≥18 years	R; X	4.5 days 3h PP Responses to last std meal assessed	LGL diet HGL diet	↓ Glucose, insulin ↑ Ghrelin ↔ Desire to eat (subj) Significant results only in White women	NR
Krog-Mikkelsen et al. [21]	29 overweight women 31 ± 7 years 27.6 ± 1.5 kg/m^2^	R; =	10 weeks 4 h PP Responses to last high or low GI breakfast assessed	LGI diet HGI diet	↓ Glucose, insulin, GLP-1 ↑ Fullness (subj) ↓ Desire to eat fatty food(sub) ↔ GLP-2, glucagon, leptin, ghrelin ↔ Ad libitum energy intake ↔ Substrate oxidation rates	No
Aston et al. [22]	19 women 34–65 years 25–47 kg/m^2^	R; X	12-week/phase No washout	LGI staples HGI staples Ad libitum	↔ Body wt, body composition, waist circumference ↔ Hunger, fullness (both subj) ↔ Energy intake	Yes
Das et al. [23]	34 overweight adults 24–42 years 25–30 kg/m^2^	R; =	12 months	LGL diet HGL diet 30% energy restriction	↔ Body wt, % Body fat ↔ Hunger, satiety (both subj)	No
Juanola-Falgarona et al. [24]	122 overweight or obese adults 30–60 years 27–35 kg/m^2^	R; =	6 months	LGI HGI HGI-LFat 500 kcal energy restriction	↔ BMI ↔ Hunger, satiety (both subj)	No

^1^ Abbreviations: GI: glycemic index; GLP: glucagon-like peptide; HDL-C: high density lipoprotein cholesterol; HFat: high fat; HGI: high GI; LDL-C: low density lipoprotein cholesterol; LGI: low GI; NR: not reported; PP: postprandial; R: randomized; subj: subjective measure; TG: triglycerides; X: crossover design; =: parallel design; ↓: lower; ↑: higher; ↔: no difference.

**Table 3:** Cross-sectional studies assessing the effects glycemic index or glycemic load on body weight ^1^.

Study	Sample	Association Trends			
		GI	GL	Fiber	CHO
Mendez et al. [29]	8195 Spanish adults 35–74 years 18.5–60 kg/m^2^	↔ BMI (men) - BMI (women)	(-) BMI	(-) GI (+) GL	(+) GL (men) ↔ GI (women) (+) GL
Hosseinpour-Niazi et al. [30]	2457 adults 19–84 years	(+) BMI ↔ WC, Glucose, total-C, LDL-C, BP (-) HDL-C (among obese) (+) TG (among obese)	↔ BMI, WC, Total-C, LDL-C, HDL-C, TG, BP (-) Glucose, 2-h glucose (among non-obese)	NR	NR
McKeown et al. [31]	2941 adults 27.2 kg/m^2^	+ Insulin ↔ Glucose	NR	NR	+ Fiber ↔ Glucose, insulin
Murakami et al. [32]	3931 Japanese women 18–20 years	+ Glucose, HbA1c, BMI	+ Glucose ↔ BMI	- BMI, GI, GL	NR
Wang et al. [33]	238 low income Latino adults w/T2D 45–67 years 33–36 kg/m^2^	↔ WC (+) HbA1c ↔ Glucose	(+) WC ↔ Glucose, HbA1c	- GI	NR
Silva et al. [34]	175 adults w/T2D 52–71 years	(+) MetS (+) WC	NR	(+) MetS (+) WC	NR
Farvid et al. [35]	640 adults w/T2D 28–75 years	↔ Glucose ↔ HbA1c	(+) Glucose (+) HbA1c ↔ BMI	(-) Glucose ↔ HbA1c ↔ BMI	(+) Glucose (+) HbA1c (when substitutes CHO for prot or fat)
Milton et al. [36]	1152 older adults (>65 years)	↔ BMI, W/H ↔ TC, LDL-C, HDL-C, TG, BP	NR	NR	NR
Castro-Quezada et al. [37]	343 rural Spanish older adults Age: 60–74 years	↔ BMI, WC	↔ BMI, WC	NR	NR

^1^ Abbreviations: BMI: body mass index; BP: blood pressure; CHO: carbohydrates; GI: glycemic index; GL: glycemic load; HbA1c: glycosylated hemoglobin; HDL-C: high density lipoprotein cholesterol; LDL-C: low density lipoprotein cholesterol; MetS: metabolic syndrome; NR: not reported; T2D: type 2 diabetes; TG: triglycerides; Total-C: total cholesterol; WC: waist circumference; W/H: waist to hip ratio; (+): positive association; (-): negative association; ↔: no association.

**Table 4:** Intervention studies with parallel design assessing the effects of chronic intake of diets with different GI on body weight ^1^.

Study	Sample	Duration	Intervention	Treatment Effects (Low vs. High GI)	Greater Fiber (Low GI)
Buscemi et al. [38]	40 obese adults 20–60 years 25–49.9 kg/m^2^	3 months	LGI diet, hypocaloric HGI diet, hypocaloric	↔ Weight loss ↔ WC ↔ BMI	No
Philippou et al. [39]	18 adults at risk for heart disease 35–65 years 27–35 kg/m^2^	12 weeks	LGI diet HGI diet Deficit 500 kcal/day	↔ Weight loss ↔ BMI	No
Abete et al. [40]	32 obese Spanish adults 36 ± 7 years 32.5 ± 4.3 kg/m^2^	8 weeks	LGI diet HGI diet 30% energy restriction	↓ Body weight	Yes
Das et al. [23]	34 overweight adults 24–42 years 25–30 kg/m^2^	12 months	LGL diet HGL diet 30% energy restriction	↔ Weight loss, % Body fat ↔ Hunger, satiety (both subj)	No
Sichieri et al. [41]	203 women 25–45 years 23–30 kg/m^2^	18 months	LGI diet HGI diet Deficit 100–300 kcal/day	↔ Weight loss	No
Juanola-Falgarona et al. [24]	122 overweight or obese adults 30–60 years 27–35 kg/m^2^	6 months	LGI HGI HGI-LFat 500 kcal energy restriction	↔ Weight loss, WC ↔ BMI ↔ Hunger, satiety (both subj)	No
Karl et al. [42]	46 overweight adults 20–42 years 25–29.9 kg/m^2^	12 months	LGL-10% energy restriction HGL-10% energy restriction LGL-30% energy restriction HGL-30% energy restriction	↔ Weight loss	No
Karl et al. [43]	91 obese adults	17 weeks	LGI-55% CHO HGI-55% CHO LGI-70% CHO HGI-70% CHO	↔ Weight loss, body composition	No

^1^ Abbreviations: BMI: body mass index; CHO: carbohydrate; GI: glycemic index; HGI: high GI; HGL: high glycemic load; LFat: low fat; LGI: low GI; LGL: low glycemic load; WC: waist circumference; ↓ lower; ↑: higher; ↔: no difference.

**Table 5:** Cross-sectional studies assessing the effects glycemic index or glycemic load on markers of glucose homeostasis ^1^.

Study	Sample	Association Trends			
		GI	GL	Fiber	CHO
Farvid et al. [35]	640 adults w/T2D 28–75 years	↔ Glucose ↔ HbA1c	(+) Glucose (+) HbA1c ↔ BMI	(-) Glucose ↔ HbA1c ↔ BMI	(+) Glucose (+) HbA1c (when substitutes CHO for prot or fat)
Wang et al. [33]	238 low income Latino adults w/T2D 45–67 years 33–36 kg/m^2^	↔ WC (+) HbA1c ↔ Glucose	(+) WC ↔ Glucose, HbA1c	- GI	NR
van Aerde et al. [45]	2078 Inuit adults 28–62 years 21–33 kg/m^2^	(+) Glucose ↔ 2 h-Glucose, HbA1c, HOMA	↔ Glucose, 2 h-Glucose, IGT, HbA1c (+) HOMA	NR	NR
Hosseinpour-Niazi et al. [30]	2457 adults 19–84 years	(+) BMI ↔ WC, Glucose, Total-C, LDL-C, BP (-) HDL-C (among obese) (+) TG (among obese)	↔ BMI, WC, Total-C, LDL-C, HDL-C, TG, BP (-) Glucose, 2-h glucose (among non-obese)	NR	NR
McKeown et al. [31]	2941 adults 27.2 kg/m^2^	(+) Insulin, TG (-) HDL-C ↔ Glucose, Total-C, LDL-C ↔ WC	NR	NR	(+) Fiber ↔ Glucose, insulin
Murakami et al. [46]	3931 Japanese women 18–20 years	(+) Glucose, HbA1c, BMI	(+) Glucose ↔ BMI	(-) BMI, GI, GL	NR
Dominguez Coello et al. [47]	668 adults 18–75 years	↔ HOMA	(+) HOMA; null when adjusted for fructose	(+) HOMA for fruit fiber (-) HOMA for cereal and vegetable fiber	(+) HOMA for fructose
Shikany et al. [48]	878 postmenopausal women 63.8 ± 7.3 years 26.9 ± 5.2 kg/m^2^	NR	(-) HDL-C (+) TG ↔ TC, LDL-C, glucose, insulin, HOMA	NR	NR
Mayer-Davis et al. [49]	1255 adults with/without IR or T2D 55.3±8.5 years 29.1±5.9 kg/m^2^	↔ Glucose	↔ Glucose, 2 h-Glucose	NR	NR

^1^ Abbreviations: BMI: body mass index; BP: blood pressure; CHO: carbohydrates; GI: glycemic index; GL: glycemic load; HbA1c: glycosylated hemoglobin; HDL-C: high density lipoprotein cholesterol; HOMA: homeostasis assessment model for insulin resistance; IGT: impaired glucose tolerance; IR: insulin resistance; LDL-C: low density lipoprotein cholesterol; NR: not reported; prot: protein; T2D: type 2 diabetes; TG: triglycerides; Total-C: total cholesterol; WC: waist circumference; (+): positive association; (-): negative association; ↔: no association.

**Table 6:** Prospective studies assessing the effects glycemic index or glycemic load on type 2 diabetes risk ^1^.

Study	Sample	F/U, y	Type 2 Diabetes Risk			
			GI	GL	Fiber	CHO
Halton et al. [54]	85,059 women	20	NR	↑	NR	↑
Villegas et al. [28]	64,227 middle-aged Chinese women	4.6	↑	↑	NR	↑
Sakurai et al. [55]	1995 adult Japanese male	6	↑	↔	↔ (total fiber)	NR
Simila et al. [56]	25,943 male smokers 50–69 years	12	↔	↔	↔	↓ (total CHO)
Barclay et al. [57]	2123 Australian adults	10	↔	NR	↔ (total fiber)	↔ (total CHO, sugar, or starch)
Mosdol et al. [58]	7321 Caucasian adults	13	↔	↑	NR	NR
Sluijs et al. [59]	37,843 Netherlands adults 21–70 years	10	↑	↑	↓	↑ (starch)
Van Woudenbergh et al. [60]	4366 Netherlands adults ≥55 years	12	↔	↔	NR	NR
Sluijs et al. [25]	16,835 adults	12	↔	↔	NR	↔
Sahyoun et al. [61]	1898 older adults 70–79 years	4	↔	↔	NR	NR

^1^ Abbreviations: CHO: carbohydrates; F/U: follow-up; GI: glycemic index; GL: glycemic load; NR: not reported; y: years ↑: increased risk; ↓: decreased risk; ↔: no difference in risk.

**Table 7:** Intervention studies with assessing the effects of diets with different GI on markers of glucose homeostasis ^1^.

Study	Sample	Design	Duration	Intervention	Treatment Effects (Low vs. High GI)	Greater Fiber (Low GI)
Botero et al. [62]	12 overweight and obese males 18–35 years 27–45 kg/m^2^	R; X	10 days/phase	LGL HGL	↓ Glucose, insulin	No
Pal et al. [18]	21 overweight and obese adults 25–65 years	R; X	21 days/phase	LGI breakfast replacement HGI breakfast replacement	↓ Glucose ↔ Insulin, HOMA	Yes
Sacks et al. [63]	163 overweight adults 53 ± 11 years 32 ± 6 kg/m^2^	R; X	5 weeks/phase	HGI-HCHO HGI-LCHO LGI-HCHO LGI-LCHO	↓ Glucose, insulin sensitivity (only with HCHO)	Yes
Shikany et al. [64]	24 overweight and obese men 34.5 ± 8.1 years 27.8 ± 3.5 kg/m^2^	R; X	4 weeks/phase	LGI/GL HGI/GL	↔ Weight, BMI ↔ Glucose, insulin ↔ CRP, IL-6, TNF-a, TNF-RII, PAI-1, Fibrinogen ↑ Total-C, LDL-C, HDL-C	No
Runchey et al. [65]	80 adults 29.6 ± 8.2 years 27.4 ± 5.9 kg/m^2^	R; X	4 weeks/phase	LGL HGL	↓ Glucose, IGF-1 ↔ Insulin, HOMA	Yes
Buscemi et al. [38]	40 obese adults 20–60 years 25–49.9 kg/m^2^	R; =	3 months	LGI diet, hypocaloric HGI diet, hypocaloric	↔ Weight loss, WC, BMI ↔ HbA1c, Glucose, HOMA	No
Jenkins et al. [66]	210 adults w/T2D HbA1c 6.5–8.0%	R; =	6 months	LGI diet High-cereal fiber diet	↓ HbA1c ↓ Glucose	Yes
Juanola-Falgarona et al. [24]	122 overweight or obese adults 30–60 years 27–35 kg/m^2^	R; =	6 months	LGI HGI HGI-LFat 500 kcal energy restriction	↔ Weight loss, WC, BMI ↔ Hunger, satiety (both subj) ↔ Glucose	No
Wolever et al. [67,68]	162 adults w/T2D HbA1c ≤ 130% of ULN 20–40 kg/m^2^	R; =	12 months	HCHO/HGI HCHO/LGI LCHO/HMUFA	↔ HbA1c, HOMA, insulinogenic index, muscle insulin sensitivity ↔ Weight loss	Yes
Pereira et al. [69]	19 healthy adults 22–38 years 27–35 kg/m^2^	R; =	45 days	LGI HGI	↔ Glucose, insulin, leptin (including AUCs) ↓ HOMA vs. baseline ↓ WC, W/H, body fat %	NR
Gomes et al. [70]	20 adults w/T2D 42.4 ± 5.1 years 29.2 ± 4.8 kg/m^2^	R; =	30 days	LGI HGI	↔ Glucose, adiponectin, CRP, total-C, LDL-C, HDL-C, TG ↑ fructosamine ↓ body weight	

^1^ Abbreviations: BMI: body mass index; CRP: C-reactive protein; GI: glycemic index; HbA1c: glycosylated hemoglobin; HCHO: high carbohydrate; HDL-C: high density lipoprotein cholesterol; HGI: high GI; HGL: high glycemic load; HMUFA: high monounsaturated fatty acids; HOMA: homeostasis assessment model for insulin resistance; IL: interleukin; IGF: insulin-like growth factor; LCHO: low carbohydrate; LDL-C: low density lipoprotein cholesterol; LFat: low fat; LGI: low GI; LGL: low glycemic load; NR: not reported; PAI: plasminogen activator inhibitor; R: randomized; subj: subjective measure; T2D: type 2 diabetes; Total-C: total cholesterol; TNF: tumor necrosis factor; ULN: upper limits for normal; WC: waist circumference; W/H: waist to hip ratio; X: crossover design; =: parallel design; ↑: higher; ↓ lower; ↔ no difference.

**Table 8:** Cross-sectional studies assessing the effects glycemic index or glycemic load on cardiovascular disease risk factors ^1^.

Study	Sample	Association Trends	
		GI	GL
Hosseinpour-Niazi et al. [30]	2457 adults 19–84 years	(+) BMI ↔ WC, Glucose, Total-C, LDL-C, BP (-) HDL-C (among obese) (+) TG (among obese)	↔ BMI, WC, Total-C, LDL-C, HDL-C, TG, BP (-) Glucose, 2-h glucose (among non-obese)
Levitan et al. [74]	18,137 women ≥45 years	(-) HDL-C (+) LDL-C, LDL/HDL, TG, CRP	(-) HDL-C (+) LDL/HDL, TG
Liese et al. [75]	1026 middle-aged adults	↔ Total-C, LDL-C, HDL-C, TG	(+) Total-C, LDL-C, TG (-) HDL-C
McKeown et al. [31]	2941 adults 27.2 kg/m^2^	(+) Insulin, TG (-) HDL-C ↔ Glucose, TC, LDL-C ↔ WC	NR
Milton et al. [36]	1152 older adults (>65 years)	↔ BMI, W/H ↔ TC, LDL-C, HDL-C, TG, BP	NR
Murakami et al. [46]	3931 Japanese women 18–20 years	(+) Glucose, HbA1c, BMI, TG ↔ TC, LDL-C, HDL-C	(+) Glucose, TG (-) HDL-C ↔ BMI ↔ TC, LDL-C
Shikany et al. [48]	878 postmenopausal women 63.8 ± 7.3 years 26.9 ± 5.2 kg/m^2^	NR	(-) HDL-C (+) TG ↔ TC, LDL-C, glucose, insulin, HOMA
Juanola-Falagrona et al. [76]	6606 adults Men: 55–80 years Women: 60–80 years	(+) MetS (among <75 years without T2D) (+) elevated TG (among 65–74 years without T2D) ↔ other MetS components	↔ MetS or its components
Castro-Quezada et al. [37]	343 rural Spanish older adults 60–74 years	↔ BMI, WC ↔ Glucose, TC, LDL-C, HDL-C, TG, BP	↔ BMI, WC ↔ Glu, TC, LDL-C, HDL-C, TG, BP

^1^ Abbreviations: BMI: body mass index; BP: blood pressure; GI: glycemic index; GL: glycemic load; HbA1c: glycosylated hemoglobin; HDL-C: high density lipoprotein cholesterol; HOMA: homeostasis assessment model for insulin resistance; LDL-C: low density lipoprotein cholesterol; MetS: metabolic syndrome; NR: not reported; T2D: type 2 diabetes; TG: triglycerides; Total-C: total cholesterol; WC: waist circumference; W/H: waist to hip ratio; (+): positive association; (-): negative association; ↔: no association.

**Table 9:** Prospective studies assessing the effects glycemic index or glycemic load on cardiovascular disease risk ^1^.

Study	Sample	F/U, y	Outcome	Type 2 Diabetes Risk			
				GI	GL	Fiber	CHO
Nagata et al. [77]	28,356 Japanese adults	16	CVD mortality	↑ (women)	↔	NR	NR
Burger et al. [78]	6192 adults with T2D	9.2	CVD mortality	↔	↔	↓	↔ CHO, sugar or starch
Levitan et al. [79]	4617 men with prior CVD 45–79 years	6	CVD mortality	↔	↔	NR	NR
Levitan et al. [80]	36,246 Swedish men 45–79 years	6	MI Stroke CVD mortality	↔ ↔ ↔	↔ ↔ ↔	NR	NR
Yu et al. [81]	117,366 Chinese adults 40–74 years	9.8 years for women 5.4 years for men	CHD	↔	↑	↑ (refined grains)	↑
Burger et al. [82]	8855 men 10,753 women 21–64 years	11.9	Stroke CHD	↑ (men) ↔	↔ ↔	NR	↔ ↑ (men; CHO, starch)
Hardy et al. [83]	13051 adults 45–64 years	17	CHD	↑ (African Americans) ↔ (when excluding participants w/T2D)	↑ (Whites) ↔ (when excluding participants w/T2D)	NR	NR
Sieri et al. [84]	44,132 adults	7.9	CHD	↔	↑ (women)	NR	↑ (women) ↔ sugar or starch
Beulens et al. [85]	15,714 Dutch women	9	CHD Stroke Combined	↑ ↔ ↑	↔ ↔ ↑	NR	NR
Levitan et al. [86]	36,234 Swedish women 48–83 years	9	MI	↔	↔	NR	NR
Levitan et al. [87]	36,019 women 48–83 years	9	HF	↔	↔	NR	NR

^1^ Abbreviations: CHD: coronary heart disease; CHO: carbohydrates; CVD: cardiovascular disease; F/U: follow-up; GI: glycemic index; GL: glycemic load; HF: heart failure; MI: myocardial infarction; NR: not reported; T2D: type 2 diabetes; y: years; ↑: increased risk; ↓: decreased risk; ↔: no difference in risk.

**Table 10:** Intervention studies assessing the effects of diets with different GI on cardiovascular disease risk factors ^1^.

Study	Sample	Design	Duration	Intervention	Treatment Effects (Low vs. High GI)	Greater Fiber (Low GI)
Botero et al. [62]	12 overweight and obese males 18–35 years 27–45 kg/m^2^	R; X	10 days/phase	LGL HGL	↓ Glucose, insulin ↔ BP, Total-C, HDL-C, TG, CRP	No
Neuhouser et al. [89]	80 overweight or obese adults 18–45 years 27.5 ± 5.9 kg/m^2^	R; X	4 weeks/phase	LGL HGL	↓ CRP (if high body fat mass) ↔ Leptin, adiponectin	Yes
Sacks et al. [63]	163 overweight adults 53 ± 11 years 32 ± 6 kg/m^2^	R; X	5 weeks/phase	HGI-HCHO HGI-LCHO LGI-HCHO LGI-LCHO	With HCHO: ↓ Glucose, insulin sensitivity ↑ LDL-C ↔ HDL-C, TG, BP With LCHO: ↔ Glucose, insulin, LDL-C, HCL-C, TG, BP	Yes
Shikany et al. [64]	24 overweight and obese men 34.5 ± 8.1 years 27.8 ± 3.5 kg/m^2^	R; X	4 weeks/phase	LGI/GL HGI/GL	↔ Weight, BMI ↔ Glucose, insulin ↔ CRP, IL-6, TNF-a, TNF-RII, PAI-1, Fibrinogen ↑ Total-C, LDL-C, HDL-C	No
Pal et al. [18]	21 overweight and obese adults 25–65 years	R; X	21 days/phase	LGI breakfast replacement HGI breakfast replacement	↓ Glucose ↔ Insulin, HOMA ↔ TG, LDL-C, HDL-C	Yes
Buscemi et al. [38]	40 obese adults 20–60 years 25–49.9 kg/m^2^	R; =	3 months	LGI diet, hypocaloric HGI diet, hypocaloric	↔ Weight loss, WC, BMI ↔ HbA1c, Glucose, HOMA	No
Philippou et al. [39]	13 adults 35–65 years 27–35 kg/m^2^	R; =	12 weeks	LGL HGL	↑ Weight loss ↓ Glucose AUC ↔ Total-C, LDL-C, HDL-C, TG, Glucose ↔ WC, Body Fat %	No
Philippou et al. [90]	38 men with high CHD risk 35–65 years 27–35 kg/m^2^	R; =	6 months	LGL HGL	↓ Insulin, HOMA ↓ TC ↔ BP, LDL-C, HDL-C, TG	No
de Rougemont et al. [92]	38 French adults 20–60 years 25–30 kg/m^2^	R; =	5 weeks	LGI starchy foods HGI starchy foods	↓ Body weight, BMI ↓ TC, LDL-C	Yes
McMillan-Price et al. [93]	129 overweight and obese young adults 18–40 years 25–30 kg/m^2^	R; =	12 weeks	HGI/HCHO LGI/HCHO HGI/HProt LGI/HProt (All LFat, HF)	↑ LDL-C w/HighGI-Hprot ↔ weight, HDL-C, TG, FFA, Glucose, Insulin, HOMA, CRP	No
Gogebakan et al. [91]	773 overweight or obese adults 18–65 years 27–45 kg/m^2^	R; =	6 months (after initial weight loss phase)	LGI/LProt HGI/HProt LGI/HProt HGI/Lprot	↔ Glucose ↓ hsCRP	No

^1^ Abbreviations: AUC: area under the curve; BMI: body mass index; BP: blood pressure; CRP: C-reactive protein; CHD: coronary heart disease; FFA: free fatty acids; GI: glycemic index; HbA1c: glycosylated hemoglobin; HCHO: high carbohydrate; HDL-C: high density lipoprotein cholesterol; HF: high fiber; HGI: high GI; HGL: high glycemic load; HOMA: homeostasis model assessment for insulin resistance; HProt: high protein; IL: interleukin; LCHO: low carbohydrate; LDL-C: low density lipoprotein cholesterol; LFat: low fat; LGI: low GI; LGL: low glycemic load; LProt: low protein; PAI: plasminogen activator inhibitor; R: randomized; Total-C: total cholesterol; TG: triglycerides; TNF: tumor necrosis factor; WC: waist circumference; X: crossover design; =: parallel design; ↑: higher; ↓ lower; ↔ no difference.
